# The Taming of *Psidium guajava*: Natural and Cultural History of a Neotropical Fruit

**DOI:** 10.3389/fpls.2021.714763

**Published:** 2021-09-28

**Authors:** Edna Arévalo-Marín, Alejandro Casas, Leslie Landrum, Myrtle P. Shock, Hernán Alvarado-Sizzo, Eduardo Ruiz-Sanchez, Charles R. Clement

**Affiliations:** ^1^Instituto de Investigaciones en Ecosistemas y Sustentabilidad (IIES), Universidad Nacional Autónoma de México, Morelia, Mexico; ^2^Posgrado en Ciencias Biológicas, Unidad de Posgrado, Universidad Nacional Autónoma de México, Mexico City, Mexico; ^3^Natural History Collections, School of Life Sciences, Arizona State University, Tempe, AZ, United States; ^4^Programa de Antropologia e Arqueologia, Instituto de Ciências da Sociedade, Universidade Federal do Oeste do Pará, Santarém, Brazil; ^5^Laboratorio de Biogeografía y Sistemática, Departamento de Biología Evolutiva, Facultad de Ciencias, Universidad Nacional Autónoma de México, Ciudad Universitaria, Mexico City, Mexico; ^6^Departamento de Botánica y Zoología, Centro Universitario de Ciencias Biológicas y Agropecuarias, Universidad de Guadalajara, Zapopan, Mexico; ^7^Coordenação de Tecnologia e Inovação, Instituto Nacional de Pesquisas da Amazônia, Manaus, Brazil

**Keywords:** guava, semi-domesticated, center of origin of domestication, archeology, genetic analyses, dispersal

## Abstract

Guava (*Psidium guajava* L., Myrtaceae) is a Neotropical fruit that is widely consumed around the world. However, its evolutionary history and domestication process are unknown. Here we examine available ecological, taxonomic, genetic, archeological, and historical evidence about guava. Guava needs full sunlight, warm temperatures, and well-distributed rainfall throughout the year to grow, but tolerates drought. Zoochory and anthropochory are the main forms of dispersal. Guava’s phylogenetic relationships with other species of the genus *Psidium* are unclear. A group of six species that share several morphological characteristics are tentatively accepted as the *Psidium guajava* complex. DNA analyses are limited to the characterization of crop genetic diversity within localities and do not account for possible evolutionary and domestication scenarios. A significant amount of archeological information exists, with a greater number and older records in South America than in Mesoamerica, where there are also numerous historical records. From this information, we propose that: (1) the guava ancestor may have originated during the Middle or Late Miocene, and the savannas and semi-deciduous forests of South America formed during the Late Pleistocene would have been the most appropriate ecosystems for its growth, (2) the megafauna were important dispersers for guava, (3) dispersal by humans during the Holocene expanded guava’s geographic range, including to the southwestern Amazonian lowlands, (4) where its domestication may have started, and (5) with the European conquest of the Neotropics, accompanied by their domestic animals, new contact routes between previously remote guava populations were established. These proposals could direct future research on the evolutionary and domestication process of guava.

## Introduction

**FIGURE 1:**
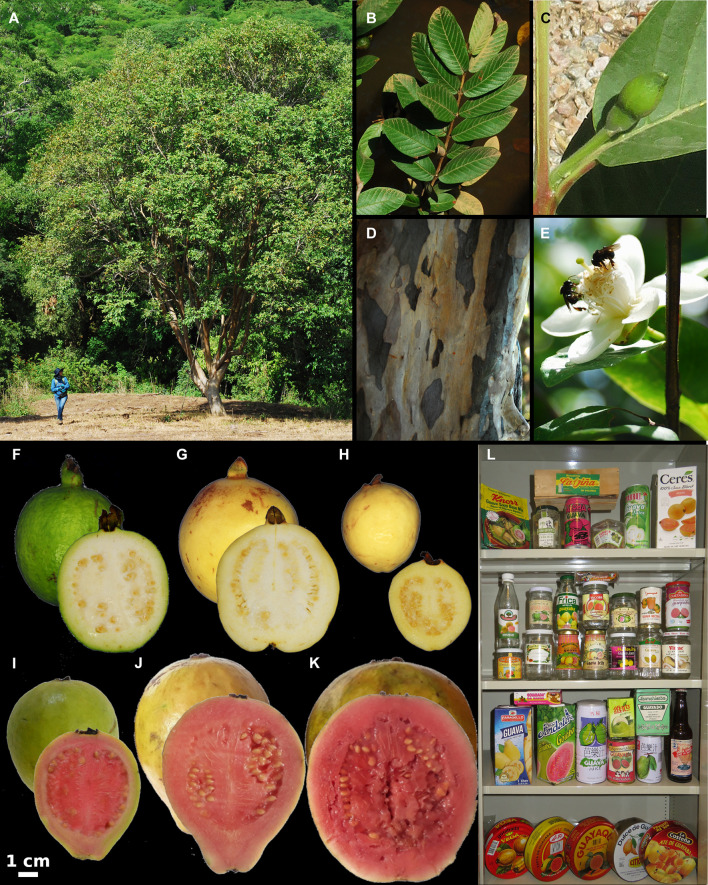
**(A)** Aspect of a feral, unpruned, guava tree at a cropland limit in Morelos, Mexico. **(B)** Typical guava branchelet with dusty leaves highlighting the venation in Morelos, Mexico. **(C)** Flower bud. **(D)** Detail of the exfoliating bark, cultivated specimen in Aguascalientes, Mexico. **(E)** Flower with a pair of hymenoptera visitors. **(F–H)** The most common variety in Central Mexico ‘media china’ and its variation. **(I)** Commercial variety in João Pessoa, North East Brazil. **(J,K)** Two commercial varieties in Bogotá, Colombia. All fruits at the same scale (0.5×). **(L)** Sample of guava products from around the world. Guava products are produced and sold locally or internationally. This sample includes products from Mexico, Central America, South America, Africa, Asia, Malaysia, Philippines, Polynesia, the Caribbean, and Middle East. They come in the form of juices, jams, jellies, pastes, pieces of fruits in syrup, soup base, dried fruits, medicinal leaf teas, and candies. Collection held by Arizona State University Herbarium. Photo credits: H. Alvarado-Sizzo **(A,B,D–H)**, L. Landrum **(C,L)**, J. Romero-Murcia **(J,K)**, and M. F. M. Brito **(I)**.

Guava (*Psidium guajava* L.) is an important perennial fruit tree who’s distribution extends from Mexico and the Antilles to Argentina and Uruguay (Landrum, 2017). It is valued for fresh consumption because of the fruits’ aroma and sweet flavor, but many of its products (pulp concentrate or jelly) have export potential (Figure 1). The consumption of guava fruits around the world and their medicinal properties have made it the most important among the minor tropical fruits, with an estimated world production of 2.3 million tons per year between 2015 and 2017, and the highest production in India, followed by China, Mexico, Egypt, and Brazil (Altendorf, 2018, 2019). Its chemical properties – vitamins C, A, B1, and B2, calcium, phosphorus, lycopene, and phenolic compounds – along with low cultivation costs and tolerance to soil salinity (Gutiérrez et al., 2008; Rajan and Hudedamani, 2019), have also contributed to this. In numerous tropical and subtropical countries where guava is cultivated, it is often quite invasive (Richardson and Rejmánek, 2011; Landrum, 2017). This success as an invasive species is probably due to its ability to combine clonal and sexual propagation during invasion events (Urquía et al., 2019), along with the production of numerous seeds that can remain viable for a long time (Adhiambo et al., 2019).

Even though guava is an economically important crop, its origin, evolutionary history under domestication, and dispersal throughout the Americas are mostly conjectural. Various authors have suggested northern South America (Risterucci et al., 2005; Aranguren et al., 2008b), northern Amazonia (Hastorf, 2006), or northeastern Brazil (Clement, 1999; Pearsall, 2008) as the area of origin, while others have proposed Central America (Brand, 1939; Nakasone and Paull, 1998). The evolutionary history of perennial crops has become a dominant theme of plant domestication research (Miller and Gross, 2011; Gaut et al., 2015; Spengler, 2020), but discovering the geographic origin and area of domestication of any crop, and even identifying its wild ancestor, can be a challenge. To date, no research has focused on the guava domestication process. Therefore, a review can help build a roadmap to guide future research.

We examine and systematize biological, molecular, archeological, and historical information about guava. Based on this review, we identify the main challenges that have so far limited our understanding of guava domestication, and we present five proposals that can guide future research about the following central questions that remain unanswered: (1) What are its closest relatives? (2) What is the area of origin of guava as a species? (3) What is (are) the area(s) of guava domestication? (4) How did guava reach its current distribution? (5) What role did the European conquerors and their domestic animals play in guava dispersal and gene flow?

## Natural History of *Psidium Guajava*

### Fossil Evidence of the *Psidium* Lineage

A warming climatic trend during the Paleocene/Eocene favored the presence of several now tropical lineages in South American high latitudes. Myrtaceae were a notable element in southern Patagonian floras during the early Paleogene (Panti, 2016). Four Patagonian fossils dated to 57-37 million years ago (mya) (Paleocene/Eocene) have been assigned to *Psidium* (Panti, 2016): *Psidium membranaceum* Engelhardt (1891), *P. araciforme* Berry (1938), *P. licciardoi* Hünicken (1967), and *Psidium* sp. (Panti, 2016). Despite strong similarities in lamina shape and venation pattern to present-day *Psidium* leaves, there is still uncertainty about these assignments.

A phylogenetic analysis of the tribe Myrteae (Vasconcelos et al., 2017) suggested that the most recent common ancestor of the genera *Psidium*, *Myrrhinium* Schott and *Mosiera* Small [named *Psidium* group in Vasconcelos et al., 2017 and part of the current Pimentinae subtribe in Lucas et al., 2019] dates to 25.62 mya, during the Oligocene. Today, we know that a progressive decline in temperature since the Oligocene and the subsequent climatic fluctuations during the middle-Miocene, caused the megathermal angiosperms (e.g., Myrtaceae) to migrate toward lower latitudes (Panti, 2016) from their former range at the southern tip of Patagonia.

### Systematics and Taxonomy of Guava

*Psidium guajava* (Figure 1) is a member of the Myrtaceae family that contains the pantropical tribe Myrteae (*sensu* Wilson et al., 2005), which comprises 51 genera and ∼2,500 species, including the large genus *Psidium* (Lucas et al., 2007; Govaerts et al., 2019). *Psidium*, as presently circumscribed, contains at least 60 species and perhaps as many as 100 (McVaugh, 1968; Landrum, 2017), and it is undoubtedly a Neotropical genus (Lucas et al., 2005; Landrum, 2017). The Atlantic Coastal Forest, Cerrado, and Caatinga biomes in Brazil, with ca. 50 species are recognized as the center of diversity for *Psidium* (Landrum, 2017, 2021).

Phylogenetic studies based on nuclear and plastid DNA regions have confirmed the monophyly of *Psidium* with *Myrrhinium* as its sister group (Lucas et al., 2005; Vasconcelos et al., 2017; Nadra et al., 2018; Flickinger et al., 2020). However, evolutionary relationships within the genus remain unresolved due to its high rates of diversification (Vasconcelos et al., 2017), its wide Neotropical distribution (Richardson and Rejmánek, 2011; Landrum, 2017), variable ploidy levels (Costa and Forni-Martins, 2007), and hybridization among species (Landrum et al., 1995). In this context, Landrum (2003; 2005; 2021) proposed three species complexes based on morphological, ecological, and geographical characteristics: the *Psidium salutare* complex, the *Psidium grandifolium* complex, and the *Psidium guajava* complex. The *Psidium guajava* complex is also proposed as a working hypothesis of the putative relatives of guava (Landrum, 2021).

### The Closest Relatives of Guava

**FIGURE 2:**
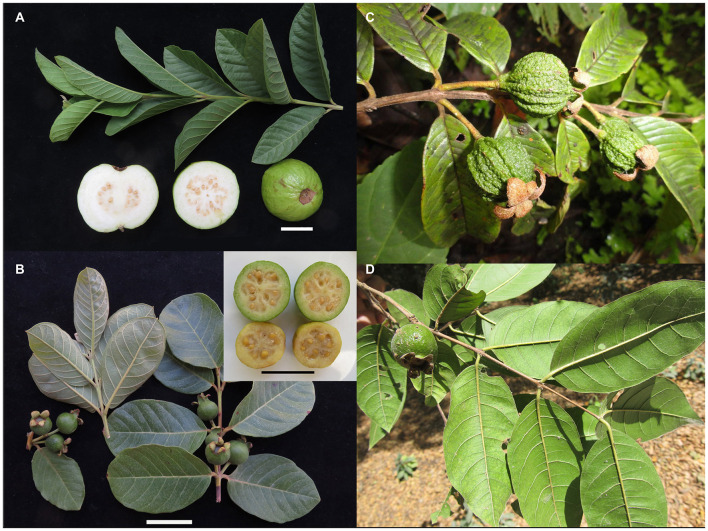
*Psidium guajava* and three other members of the *P. guajava* complex. **(A)** *P. guajava*, fruit from Lee Lee International Market, Mesa, AZ, United States, the twig and leaves grown from seed from the same variety of guava sold in same market. **(B)** *P. guineense*, cultivated plant at Tempe, Arizona, United States; grown from seeds collected in Chiapas, Mexico. **(C)** *P. rutidocarpum*, wild growing plant from Estación Biológica Huampal, Parque Nacional Yanachaga-Chemillen, Oxapama, Peru. **(D)** *P. rostratum*, twig with leaves and fruit, wild growing plant from Bosque Protector Cerro Blanco, Guayas, Ecuador. Bars all = 3 cm. Photos by **(A,C)**, L. R. Landrum **(B)**, R. Rojas **(D)**, and X. Cornejo.

**FIGURE 3:**
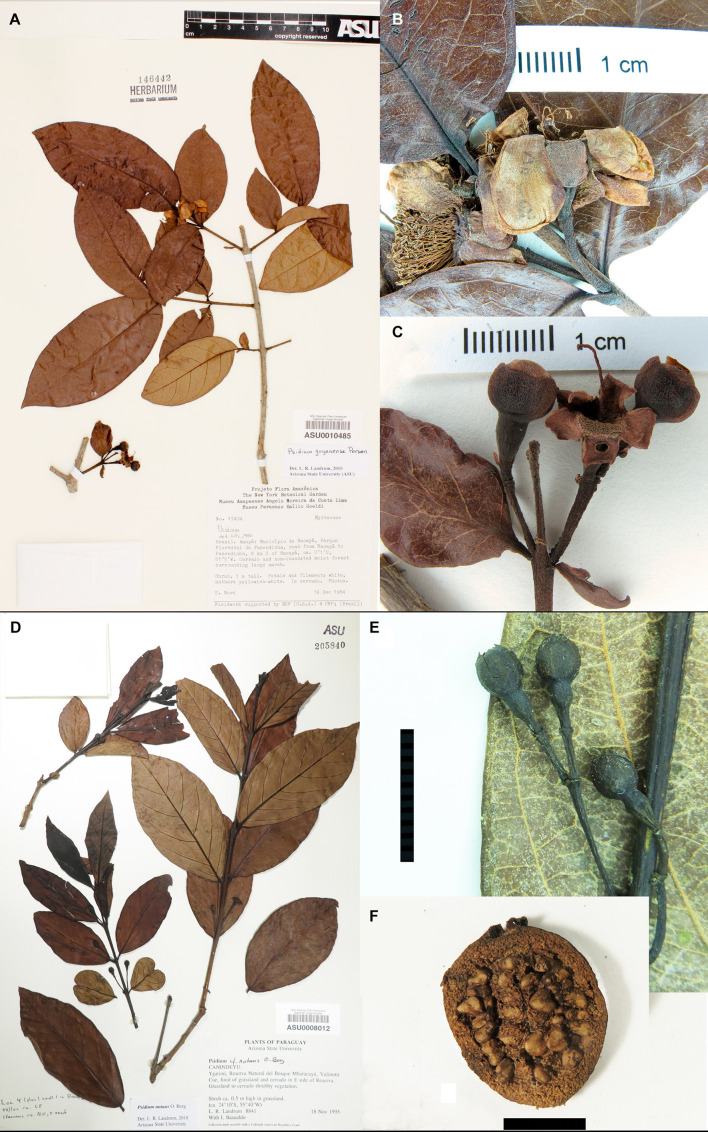
Members of the *P. guajava* complex. *Psidium guyanense*. **(A)** Herbarium sheet, showing elliptic leaves with acute to acuminate apexes. **(B)** Open flowers. **(C)** Old flower and closed flower buds showing apical pore in calyx. Outer surfaces of flowers covered with very short, erect hairs. Specimen *S. Mori 17404* from Mun. de Macapá, Amapá, Brazil. *Psidium nutans*. **(D)** Herbarium sheet, showing elliptic leaves with few lateral veins. Specimen from *Landrum & Basualdo 8841*, Canineyú, Paraguay. **(E)** Closed flower buds, the minute calyx lobes barely visible in upper left bud. Specimen from *Guillén & Choré 2426*, Santa Cruz, Bolivia. **(F)** Longitudinal section of fruit showing many small seeds. Specimen from from *Zardini & Guerrero 38496*, Misiones, Paraguay. All photos by L. R. Landrum of specimens at ASU.

**FIGURE 4:**
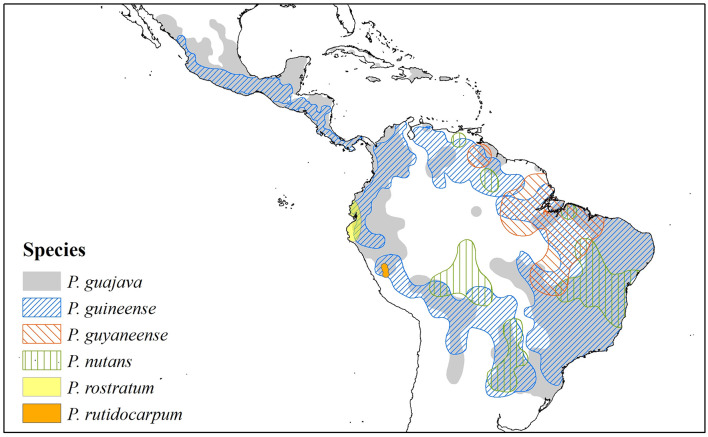
Map with the current distribution of the six species of the *Psidium guajava* complex based on geo-referenced records available in the Global Biodiversity Information Facility (GBIF), the Botanical Information and Ecology Network (BIEN), the Integrated Digitized Biocollections (IDigBio), Species Link, and The Cooperative Taxonomic Resource for American Myrtaceae (CoTRAM). The distribution of *P. guajava* is certainly more extensive than shown, because botanists and ethnobotanists don’t always collect common cultivated species.

The *Psidium guajava* complex (Figures 2, 3) is conceived as a morphological cluster of species that have similarities in their characteristics (states) that make them distinct from other species of the genus. There are no unique characteritics that define the group, but rather they share a combination of characteristics, such as venation pattern, seed number and size, indumentum pattern, and calyx structure. The complex as presently conceived includes *Psidium guajava* L., *P. guineense* Sw., *P. guyanense* Pers., *P. nutans* O. Berg., *P. rostratum* McVaugh, and *P. rutidocarpum* Ruiz & Pav. (Figures 2, 3 and Supplementary Data 1). The geographic distribution of the complex extends from Mexico to northern Argentina and Uruguay, including the Antilles (Figure 4). A molecular phylogenetic analysis shows *P. guajava* and *P. guineense* as sister taxa (Salywon, 2003), but the relationships with the other species are unclear due to lack of representation (Rivero et al., 2012; Nadra et al., 2018).

Within the complex, *Psidium guajava* and *P. guineense* (Figures 2A,B, respectively) are “weedy” species that frequently grow together and are sometimes confused with each other. *Psidium guajava* shares with *P. rutidocarpum* (Figure 2C) leaves with numerous lateral veins (not found in other species of *Psidium*). While *P. guajava* is a widespread weedy species, *P. rutidocarpum* has a restricted range in apparently natural habitats in the upper Amazon of Peru. *Psidium guineense*, *P. guyanense* (Figures 3A–C), and *P. nutans* (Figures 3D–F) are remarkably similar among themselves and could be merged into one large variable species, but they can be distinguished using the characters described in the key proposed by Landrum (2021). *Psidium rostratum* (Figure 2D) is geographically separated from other members of the *P. guajava* complex and only shares the characteristics of flower size (long style, numerous stamens), closed calyx, and placenta, but differs from other species in having fewer ovules and a few large seeds; its venation is similar to *P. guineense*, which justifies its tentative assignment to this complex.

### Habitat, Distribution, Dispersers, and Ecology of Guava

**FIGURE 5:**
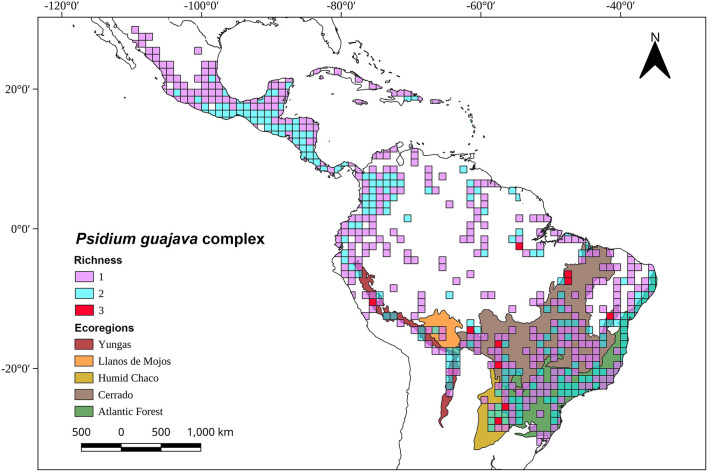
Map with species richness of the *Psidium guajava* complex by one-degree grids and selected South American ecoregions. The geo-referenced records were obtained from the following databases: the Global Biodiversity Information Facility (GBIF), the Botanical Information and Ecology Network (BIEN), the Integrated Digitized Biocollections (IDigBio), Species Link, and The Cooperative Taxonomic Resource for American Myrtaceae (CoTRAM).

Current habitats of *P. guajava* and *P. guineense* are mainly disturbed areas, such as roadsides, grasslands, and orchards, from near sea level to 1600 m (Landrum, 2017). Their distributions extend from Mexico and the Antilles to northwestern Argentina (Figure 4). *Psidium nutans* is found primarily in humid grasslands or riparian habitats at elevations from 150 to 750 m in Argentina, Bolivia, Brazil, Paraguay, and Venezuela (Landrum, 2017). *Psidium rutidocarpum* is an endemic species of the Peruvian Yunga (500 to 2300 m) and open Amazonian forest (80 to 500 m) (Kawasaki and Holst, 2006), while the natural habitat of *P. rostratum* is the Equatorial dry forest located in northwestern Peru and southwestern Ecuador (Linares-Palomino et al., 2010). *Psidium guyanense* has been reported in moist forests in the Amazon biome (Guayana region and Brazil) and the Brazilian Cerrado (Conceição and Aragão, 2010; Figure 4). An exploratory richness analysis (Figure 5) identified savannas and yungas as high species richness areas for the *P. guajava* complex. Nevertheless, we ruled Yungas vegetation out as suitable for *P. guajava* because this region is rich in dense, layered vegetation, typical of montane forests, which prevents the direct light necessary for guava development.

Most *Psidium* fruits are fleshy berries that have a strong sweet scent and are externally yellow or green. Today, guava seeds may be dispersed effectively by birds and mammals (e.g., bats, monkeys, ungulates, marsupials) (Gressler et al., 2006; Torres and Gutiérrez, 2018). Some studies have shown that domestic ungulates can disperse between 18,000 and 49,000 guava seeds per day during the fruiting peak (Somarriba, 1985, 1986; Herrera, 2013). Although Perissodactyla (horses and tapirs) are far more likely to disperse and consume sugary fruits than true ruminants (Spengler, 2019), cows are enthusiastic guava consumers (Somarriba, 1985). Double digestion of Artiodactyla presumably does not affect guava seed germination, but seed losses occur due to chewing (Somarriba, 1986). The morphological traits of guava and its abundant fruit production suggest that this tree probably had more efficient dispersers, such as Pleistocenic megafauna, capable of dispersing large quantities of seeds over long distances. Megafaunal fruits are characterized by having seeds that can escape dental grinding by large mammals (one or two large seeds or numerous small seeds) and present dull coloration (brown, yellow, or green). Janzen and Martin (1982) defined this as the Megafaunal dispersal syndrome and guava appears to fit the description.

Guava is a heliophyllic species. Its seeds need a long photoperiod (>10 h) with temperatures ranging between 20 and 30°C to attain > 90% germination (Sugahara and Takaki, 2004). Exposure of mature trees to direct sunlight guarantees abundant fruiting with increased branch growth (Paull and Duarte, 2012). The highest fruit yields are at mean air temperatures of 23 to 28°C (Menzel, 1985). These temperatures are also adequate for the germination of *P. guineense* seeds (Mugnol et al., 2014). Guavas are drought-resistant and well-adapted to different rainfall conditions, although optimal growth and fruit yield occur with 1,000 to 2,000 mm of rainfall well-distributed throughout the year (Menzel, 1985). They are sensitive to cold and are killed or severely injured by prolonged temperatures below freezing, but they can withstand light frosts. Guava adapts to various soil types, although it prefers deep, fertile, well-drained loamy, or sandy-clay soils (Menzel, 1985). Trees survive floods and can grow in seasonally waterlogged soils.

### Reproductive Biology and Pollination

Research on breeding systems in *Psidium* focuses primarily on *P. guajava*, which is an allogamous species characterized by an open pollination reproductive cycle (Torres and Gutiérrez, 2018). Nevertheless, self-pollination can occur. Bee-pollination, in which pollen is the sole reward, is the dominant pollination system in *Psidium* (Figure 1C; Proença and Gibbs, 1994; Lughadha and Proença, 1996; Gressler et al., 2006). Species of the superfamily Apoidea are important pollinators of both *P. guajava* and *P. guineense* (Lughadha and Proença, 1996; Boti, 2001; Alves and Freitas, 2007; de Siqueira, 2012; Ramos et al., 2019). Both species are also pollinated by the European honeybee, *Apis mellifera* (Soubihe Sobrinho and Gurgel, 1962; Boti, 2001; Gressler et al., 2006; Pommer and Murakami, 2009). *Psidium guyanense*, *P. nutans*, *P. rostratum*, and *P. rutidocarpum* lack comprehensive studies of their breeding systems, pollination, and dispersal.

### Cytogenetics and Hybridization

The basic chromosome number for Myrtaceae is x = 11 (Atchison, 1947). Usually, this family shows little variation in chromosome number, with 2n = 22 in most genera, but some studies have reported polyploidy in *Psidium* (Costa et al., 2008; Tuler et al., 2019). Guava karyotype analyses show a predominance of 2n = 2x = 22 chromosomes (Supplementary Table 1), with five metacentric (pairs 3, 4, 8, 9, and 10) and six submetacentric chromosomes (pairs 1, 2, 5, 6, 7, and 11) (Coser et al., 2012; Marques et al., 2016). The genome size is small (2C = 0.95 pg; Supplementary Table 1), following the classification suggested by Soltis et al. (2004). Variable levels of ploidy have been reported, mainly associated with polyploidy in cultivated plants (Supplementary Table 1).

The karyotype of *P. guineense* has 2n = 4x = 44 chromosomes (Chakraborti et al., 2010; Souza et al., 2015; Marques et al., 2016; Tuler et al., 2019), with mainly metacentric (11, 12) and submetacentric (1–10, 13–22) chromosomes (Marques et al., 2016).

Hybridization events between *P. guajava* and *P. guineense* were reported by Landrum et al. (1995). Using macro-, micro-morphological and chemical characteristics, they found intermediate individuals, which exhibit a combination of traits from both species. Molecular analyses are necessary to corroborate introgression between these species, backcrossing, and probable F1 hybrids.

### Genetic Insights

Guava genetic studies have used different molecular markers to characterize genetic diversity in feral individuals, germplasm collections, and cultivars, inside and outside its native distribution (Supplementary Table 2). These characterizations aimed to identify genotypes of interest for developing breeding programs, which can be useful to understand the domestication of guava, although the characteristics of the different markers used are not easily comparable. For this reason, and because of its overall performance (Selkoe and Toonen, 2006), we only considered the data obtained using microsatellites (simple sequence repeats – SSR).

More than 300 specific microsatellite markers have been developed for guava (Risterucci et al., 2005; Guavamap, 2008), which have been used in studies for guava genetic characterization in several countries (Supplementary Table 3). Within the range of its natural distribution, the Mexican germplasm bank, which includes both feral and cultivated samples, showed slightly higher values of diversity (H_*e*_ = 0.75; Sánchez-Teyer et al., 2010), than those obtained from Venezuelan wild samples (H_*e*_ = 0.73; Aranguren et al., 2008a). Outside of its native Neotropical distribution (Asia and the United States), guava germplasm banks (Viji et al., 2010; Mehmood et al., 2016; Urquía et al., 2019) showed similar levels of genetic diversity (Supplementary Table 3), while Asian crops and collections presented both high and low diversity values (Kanupriya et al., 2011; Chaithanya et al., 2014; Sitther et al., 2014; Kherwar et al., 2018; Kumari et al., 2018).

Most of the studies reviewed here showed low observed heterozygosity values compared to expected heterozygosity (Supplementary Table 3). Inbreeding or founder effects could explain this pattern (Motamayor et al., 2002; Erfani et al., 2012). Evolutionary genetic studies that explore the geographical origin and genetic diversity of the guava throughout the range of its natural distribution are necessary to understand the guava domestication process and dispersal routes.

## Cultural History of *Psidium Guajava*

### Archeological Evidence

**FIGURE 6:**
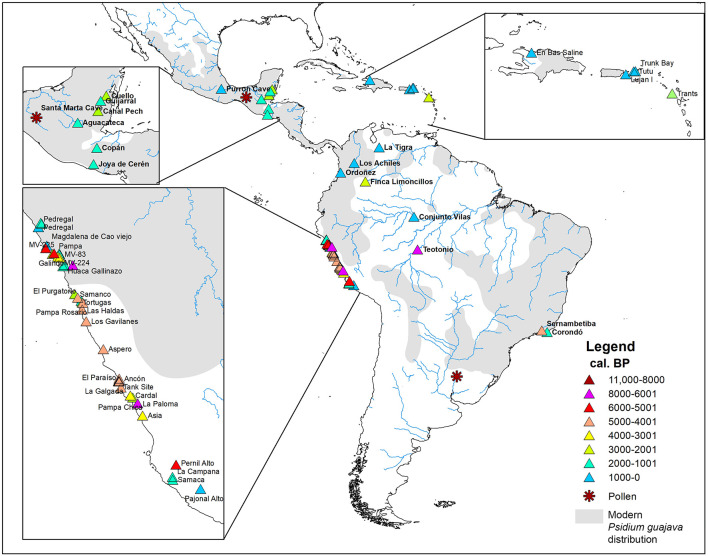
Map showing the location of the archeological sites with records of *Psidium guajava* in the Americas. Stars and triangles represent the archeological sites where micro and macro-remains were found, respectively. Colors indicate the calibrated age ranges according to Supplementary Table 4.

Fruit trees were important resources for the pre-Columbian inhabitants of the Neotropics (Piperno and Pearsall, 1998; Clement et al., 2021). Along the Peruvian coast, archeological sites with guava macro-remains date back to 7.000 calibrated years before the present (cal. BP) (Supplementary Table 4 and Figure 6) (Cárdenas, 1999). According to Hastorf (2006), guava arrived on the Peruvian coast from northwestern Amazonia via the low gap in the Andes of Ecuador, while for Pearsall (2008) crops like guava, peanut, manioc, and gourd were introduced from the eastern lowlands by way of the Andes. Later, by ca. 5400 cal. BP guava occurs in archeological sites located on the northern, central, and southern Peruvian coast (Supplementary Table 4).

The earliest evidence of guava in northern South America (Colombia), the Antilles (Monserrat), and Mesoamerica (Belize) date to ca. 2600 cal. BP, from the sites at Finca Limoncillos, Trants, and Cuello, respectively (Supplementary Table 4 and Figure 6). This chronology may be coincidental, due to the archeological sample, or a product of rapid human dispersal of the plant. However, it is suggestive of dispersal occurring only millennia after the widespread establishment of guava on the Pacific coast. Most of the sites in these regions date to the last 1500 years, suggesting later widespread utilization of the fruit.

The dry tropical forests of Mesoamerica provide a different scenario. The only remains recovered thus far are four fruit fragments dating to ca. 670 cal. BP from Purron cave (Palo Blanco phase) in the Tehuacán Valley (Figure 6) and may coincide with the period in which the area was under intensive cultivation with irrigated fields (Smith, 1967). Smith (1967) interprets the late introduction of guava as it being a luxury food, one of a group of fruits that were not grown before the establishment of an irrigation system that permitted the cultivation of enough staples in this dry environment.

Recent research in Brazilian Amazonia could push this date back from the earliest coastal Peruvian remains, with a *Psidium* seed from the Teotonio archeological site (Figure 6), near Porto Velho (state of Rondonia), in a layer dated to between 9490 and 6505 cal. BP (Supplementary Table 4). In this context Watling et al. (2018) also found carbonized seeds of other perennial food species, such as Brazil nut (*Bertholletia excelsa* Humb. & Bonpl.), pequiá (*Caryocar* sp.), and micro-remains (phytoliths) of léren (*Calathea* sp.). These findings give insights about food production in southwestern Amazonia, and are also considered evidence of disturbed areas around the human occupation. Guava is a hardy plant and highly adaptable to disturbed habitats, which explains its presence near areas of dense Amazonian vegetation.

Even older evidence comes from pollen remains recovered in the Pay Paso-1 site, near the Cuareim River in Uruguay (Figure 6), dated to ca. 11,735 cal. BP (Suárez, 2018). This suggests that *Psidium* trees were part of riverside vegetation and were available for human groups that occupied the Cuareim River region during the Pleistocene-Holocene transition (Suárez, 2018). In Mesoamerica, specifically in the Santa Marta cave (Chiapas, Mexico), Acosta et al. (2013, 2018) also recovered pollen grains, some of them tentatively attributed to *Psidium* sp., dated to the same millennia as the Uruguayan remains (Figure 6 and Supplementary Table 4).

We consider that the pollen remains attributed to *Psidium*, both in the Pay-Paso 1 site (Uruguay) and Santa Marta cave (Mexico), should be interpreted with caution. Usually, *Psidium* pollen grains are tricolpates and occasionally tetracolpates (Thornhill et al., 2012). Those reported from Santa Marta Cave are tetracolpate, apparently the least common form. For the Pay Paso site, there is no information on the shape of *Psidium* pollen grains. The determination of the Myrtaceae genus by pollen recovered in archeological sites using only light microscopy (LM) is notably troublesome and especially difficult at the species level (Tuler et al., 2017). Therefore, more evidence is needed to corroborate the early presence of *Psidium* in Mesoamerican and southern South American archeological sites.

### Post-Columbian Historical Evidence

In the Americas, fruits have historically represented a substantial component of indigenous people’s diets (Patiño, 1963). Curiously, guava was despised by some of the first Europeans who arrived in the New World. For instance, a Dominican Friar wrote “It [guava] stinks like a bug, and it was an abomination to eat it” (Rodríguez Demorizi, 1942). But later, its medicinal and nutritional values were recognized and then encouraged beyond the Americas. Guava historical records from México and the Caribbean date back to the 16th century. The official historian of the West Indies, Gonzalo Fernández de Oviedo y Valdés, reported the presence of guava fruits during the Yucatán expedition in 1517 (Patiño, 2002). Later, Francisco Cervantes de Salazar, who chronicled the Conquest of Mexico, first mentioned guava for its medicinal use to treat diarrhea (Cervantes de Salazar, 1971; Patiño, 2002). Differences between continental and Caribbean guava fruits were also recorded early by Bartolomé de las Casas: the fruits found in the Dominican Republic were smaller than those grown on the mainland (Patiño, 2002). The Licentiate Echagoian, judge for the Audience of Santo Domingo, considered guava to be a plague (Rodríguez Demorizi, 1942; Patiño, 2002). In Puerto and Santo Domingo, some chroniclers recorded guava as a potent weed in cattle pastures (Patiño, 2002).

In the expeditions which explored western El Nuevo Reino de Granada in 1553, Pedro Cieza de León (conqueror, chronicler, and historian) described the abundance of good quality guavas used for stomach illnesses in Colombia and the Darién Gap (Patiño, 2002). In 1526, Oviedo y Valdés reported that indigenous people from Darien, current Panama, distinguished between domestic and spontaneous guava trees. He also highlighted the quality of the wood and a guava variety with red-white flesh (de Vedia, 1852). Friar Pedro Aguado recorded, in the central region of the El Nuevo Reino de Granada, the consumption of pink guavas and registered the guava wood use in domestic activities (Aguado, 1917; Patiño, 2002). Reports of guava throughout the El Nuevo Reino de Granada (current Colombia, mainly) were included by several chroniclers in their descriptions of the trees and fruits used by indigenous people between 1571 and 1743 (Patiño, 2002). In Venezuela, the Spaniards found guavas in the municipalities of Lagunillas and Mérida, as recorded by Friar Pedro (Aguado, 1917). Guava trees also were reported by several chroniclers between 1535 and 1743 along the Orinoco River (Patiño, 2002).

Diego de Trujillo, Spanish chronicler and conqueror, in 1530-1533, observed guavas at several places along the Ecuadorian coast during the expedition that ended with the conquest of the Inca Empire (Patiño, 2002). Around the same time, guava crops were reported near the city of Cuzco in 1534 and north coast of Peru (1535 and 1630) (Patiño, 2002). Different kinds of guava with various colors, shapes, and sizes were found during expeditions through Peru and Ecuador in the mid and end of the 16th century (Patiño, 2002).

The chronicler Fray Gaspar de Carvajal observed guava trees in the Amazon Valley near the Andes in 1541–1542. Likewise, guavas were reported along the Manu River expedition in 1567–1569. The Oviedo y Valdés chronicles from the 16th century reported the use of guava by the Guarani in southeastern and southern Brazil and Paraguay (Patiño, 2002). Guava was also recorded in Bahia, Pernambuco, and Paraíba states (northeastern Brazil) by Portuguese chroniclers between 1582 and 1618 (Patiño, 2002).

## Five Proposals to Guide Future Research

This review of the natural and cultural histories of guava in the Neotropics provides information that can inform future research. We propose that: (1) guava originated in the savannas and semi-deciduous forests of South America; (2) megafauna were important dispersers of guava; (3) guava domestication started in southwestern Amazonia; (4) subsequent expansion of guava’s geographic range is the outcome of human migrations during the pre-Columbian times; and (5) European conquest and the introduction of domestic animals (exotic megafauna) in post-Columbian times promoted secondary contact among guava populations.

### Proposal 1: Guava (*Psidium guajava*) Originated in the Savannas and Semi-Deciduous Forests of South America

A common characteristic among species of the *Psidium guajava* complex is their affinity for open forests habitats. Guava plants, in particular, flourish in full sunlight, warm temperatures, rainfall well-distributed throughout the year, and well-drained soils. These ecological characteristics are present, with their respective variations, in the tropical savanna and tropical deciduous forest biomes, where several of the species of the complex are currently distributed.

Due to a drop in temperature since the Eocene, some Myrtaceae lineages’ distributions shifted from Patagonia toward tropical zones during subsequent epochs. Thus, the lineage from which the current genus *Psidium* derived possibly required warmer climatic conditions and open forests, which were only present from the Middle or Late Miocene. Reconstructions of the distribution of biomes in the later Miocene, using palaeoecological data and model-predicted vegetation, show evidence for a warmer world than at present and the emergence of tropical savanna and tropical deciduous forest in the center and east of South America (Pound et al., 2011). Tropical savannah covered a large part of the current areas of the humid Chaco and the Cerrado (see Figure 6 in Pound et al., 2011). Palaeobotanical studies showed fossil evidence of woody species related to the current plant taxa of the Chaco and Cerrado, which date from the Late Miocene. According to Anzótegui et al. (2019), the palaeoflora that gave rise to the current Chaco and Cerrado vegetation could have diversified earlier, in the Middle Miocene, and the fossil ancestors of Cerrado vegetation could have had a broader and more southern geographical distribution.

With this in mind, and the analysis of *P. guajava* complex species (Figure 5), we propose that the guava ancestor originated during the Middle or Late Miocene in savanna-like vegetation. Later, the Humid Chaco and the Cerrado ecoregions formed during the Pleistocene, would have presented the most suitable ecological conditions for the diversification of the species of *Psidium* that prefer open forests. We suggest the Humid Chaco and/or Cerrado are likely the areas of origin for *P. guajava*. Phylogenetic and biogeographic analyses will be useful to determine the relationships among *Psidium* species, especially within the *P. guajava* complex, to unravel its diversification and dispersal processes. They will also be important to support or refute our proposal for the center of origin of guava (*P. guajava*).

### Proposal 2: Megafauna Disperse Guava Into Other Suitable South American Ecosystems

The large fleshy fruits (4 to >10 cm diameter, details in Guimarães et al., 2008) of many tree species, including guava, share a set of traits inconsistent with current seed dispersers (Janzen and Martin, 1982). Species with large fleshy fruits, numerous seeds and abundant sugars could be the outcome of mutualistic relationships of plants with now-extinct animals, primarily with Pleistocene megafauna (Janzen and Martin, 1982; Jordano, 1995; Guimarães et al., 2008).

Reconstructions of ancient diets and habitat preferences of Pleistocene South American mega-omnivores through stable isotope analyses revealed that these animals’ subsistence consisted of C3 plants, or a mixture of C3 and C4 plants (MacFadden, 2005; Dantas et al., 2013). Since, from a functional and evolutionary perspective, fleshy fruits are usually accepted as adaptations for animal dispersal (Spengler, 2019), in South America fruit-trees could have been a C3 food source relevant to the megafauna’s diets (Janzen and Martin, 1982; Donatti et al., 2007; Guimarães et al., 2008). The evolution of large fruits in *Psidium guajava* with high sugar content was likely accompanied by strong selection pressures for seed dispersal, which led to the recruitment of large animals (Spengler, 2020).

Megafauna are effective long-distance dispersers as they depend on the vast extensions of open spaces and grassland to survive (De Vivo and Carmignotto, 2004). South American savannas were the habitat of *Toxodon*, *Glyptodon*, *Holmesina*, *Pampatherium*, *Megatherium*, *Stegomastodon*, *Tapirus*, and *Equus* (Carlini et al., 2004; Ríos et al., 2014). Paleogeographic evidence has demonstrated similarities in the taxonomic composition of the megafauna in the savannas of Bolivia, Paraguay, Argentina, and the faunal associations found in the tropical areas of Brazil. This suggests migratory phenomena and faunal exchanges between savanna ecosystems and tropical open forests, which consequently would have allowed the dispersal of plants (Carlini et al., 2004; Ríos et al., 2014). Pires et al. (2018) proved that the long-distance dispersal of seeds by gravigrade species could be up to ten times greater than that of current smaller-sized mammals, favoring gene flow and the ability of these species to adapt (Spengler, 2020).

In the case of guava, it has been demonstrated that ungulates, especially domestic ones, can consume and disperse many fruits and seeds of guava. In this perspective, we propose that guava’s distribution was expanded by large omnivores within the Humid Chaco or the Cerrado, and colonized open areas in the Atlantic Forest, the Beni savannas (Llanos de Mojos), or during humid intervals, parts of Northeastern Brazil.

### Proposal 3: Guava’s Neotropical Distribution Is the Outcome of Human Migrations in Pre-Columbian Times

Dispersal of guava by megafauna (Proposal 2), does not account for the modern geography of this fruit due to ecological and environmental restrictions, both of megafauna and guava, from thriving in tropical rainforests. Guava is widely dispersed in South America and Mesoamerica today. The archeological and historical records demonstrate that at least part of this range existed before European arrival.

Following megafauna extinction, indigenous people’s food use became the principal dispersal agent for a subset of large-fruited species. van Zonneveld et al. (2018) demonstrated that megafaunal fruits, such as the sapodilla tree (*Manilkara zapota* (L.) P. Royen), soursop (*Annona muricata* L.), and hog plum (*Spondias mombin* L.), increased or maintained their distribution ranges in the Americas through their incorporation into human diets. This suggests active human consumption and management of Neotropical fruit species and, moreover, pre-Columbian exchange, including between subcontinents (van Zonneveld et al., 2018). Dispersal and adoption of edible plants benefited from routes of movement, connection and trade between widespread human groups, both overland and by waterways. Maritime routes have facilitated the transfer of edible plants over long-distance dispersal, allowing humans to exchange and trade products between regions separated by hundreds of kilometers.

When examining the archeological occurrence of guava, we observe remains in numerous archeological sites that fall outside of its current distribution, specifically along the Pacific coastline and the interior of Amazonian forests. In both instances, the area is not optimal for its growth so the creation of additional spaces with ecological conditions favorable would have been the key to maintaining guava in new places; human settlements had these ecological conditions (Clement et al., 2021).

The archeological chronology of remains in the Antilles, with older sites in the south, suggests that the maritime introduction of guava was by way of northern South America, a hypothesis that is testable using genetic methods. The dispersal of guava into Mesoamerica could be either maritime or terrestrial or both, from the Antilles or overland from Colombia’s tropical lowlands through Central America, a route demonstrated for the spread of numerous Amazonian plants (Piperno et al., 2000; Powis et al., 2011). Geographic range expansion of *P. guajava* is likely a consequence of human migrations, exchange, and trade in pre-Columbian times, alongside the creation of additional spaces with favorable ecological conditions for guava.

### Proposal 4: Guava Domestication Began in the Lowlands of Southwestern Amazonia

Southwestern Amazonia is considered one of the most important centers of crop genetic diversity in the New World, where several native species were domesticated to some degree (Clement, 1999; Clement et al., 2015, 2016; Watling et al., 2018). The compilation of archeological evidence for guava is surprising because the earliest confirmed macrobotanical remains come from locations outside of the plant’s current range (Figure 6): the Pacific coast of Peru. For guava to occur there, necessary conditions were that (1) the plant was dispersed far beyond the expected range of Pleistocene megafaunal dispersal and (2) an appropriate ecological niche existed or was created. With respect to condition 1, humans are effective Holocene dispersers and the archeological dates support this, with the possible earliest date in SW Amazonia. With respect to condition 2, guava is an invasive plant that grows vigorously in areas disturbed by humans (Hastorf, 2006) and we can assume that it was as popular in the early Holocene and after European arrival.

The data from the Teotônio site can be used to suggest the early domestication of guava in southwestern Amazonia (Watling et al., 2018) and subsequent introduction to the Peruvian coast. However, we are approaching this hypothesis with caution as at Teotônio the evidence is sparse and there are only two dates that bracket the stratum with the “oldest” archeological remains (we have chosen to use the midpoint between the radiocarbon assays, 9490 to 6505 cal BP, for the Figure 6). Support for SW Amazonian cultivation of guava comes predominantly from secondary sources: the aforementioned importance of the region as a center of crop domestication (Clement et al., 2016) from which plants like manioc and peanut were introduced to the Pacific coast (Piperno, 2011), and the probable engagement of humans at Teotônio with cultivation of the root crop léren (Watling et al., 2018) and those in the Llanos de Mojos with léren, manioc, and squash on constructed forest islands (Lombardo et al., 2020).

### Proposal 5: The Introduction of Domestic Animals and Transfer of Guava Fruits in Post-Columbian Times Promoted Secondary Contact

Historical records indicate Europeans did not initially value the guava fruits for cultural reasons (for instance, their strong aroma). Nevertheless, guava started to gain acceptance in European communities due to the diverse sizes and colors of fruits and its medicinal benefits, which probably led to selection and later transfer of plant material.

Beyond the intense indigenous interactions during millennia described above, the European conquerors established new maritime routes for exchange between different communities and geographic areas. The contact between plant populations could have produced tree fruits with characteristics from different pre-conquest plant populations, as well as introducing new situations that could change selections desired by both indigenous peoples and new European arrivals.

European colonization also introduced domestic livestock to many areas of the Americas, especially cattle to regions where the ecological conditions were conducive to ranching. According to Janzen and Martin (1982), domestic megafauna may replicate some of the interactions between fruit trees and extinct megafauna. The introduction of such domestic animals from Europe to America provided suitable dispersers for megafaunal fruits and seeds (Janzen and Martin, 1982; Barlow, 2000). Livestock may have increased guava dispersal by favoring the establishment of the guava populations in areas where the species previously did not occur and enhancing gene flow. Given the weedy nature of guava, it may be expected that if it is dispersed into areas with suitable rainfall, light, and temperature, it would have easily established itself. European livestock, while plentiful on ranches, were also frequently maintained on small farms where some pigs and cattle were raised for domestic meat and milk products. Tracing records and the paths of livestock introduction in different regions of the Americas may contribute to interpreting the genetic history of guava, especially outside of areas where it is well represented in the archeological record.

## Final Remarks

This review of the literature about the natural and cultural history of guava offers guidance to better understand guava’s domestication and dispersal in the Neotropics. The third and fourth proposals are especially amenable to testing with modern molecular methods (genetics/genomics), although they will require extensive collaboration by a Neotropical network of researchers to collect samples and analyze them with uniform methods. A preliminary analysis is underway and will be published soon.

## Author Contributions

EA-M, AC, and CC conceived the idea. AC and CC conceptualized and supervised the project. EA-M analyzed the data and drafted the manuscript. LL conducted the taxonomic analysis. MS assisted in the collection and analysis of archeological data. HA-S and ER-S assisted in manuscript conceptualization. All authors discussed the results, contributed, and revised the final manuscript.

## Conflict of Interest

The authors declare that the research was conducted in the absence of any commercial or financial relationships that could be construed as a potential conflict of interest.

## Publisher’s Note

All claims expressed in this article are solely those of the authors and do not necessarily represent those of their affiliated organizations, or those of the publisher, the editors and the reviewers. Any product that may be evaluated in this article, or claim that may be made by its manufacturer, is not guaranteed or endorsed by the publisher.
