# Supplementary material for: The Taming of Psidium guajava: Natural and Cultural History of a Neotropical Fruit
Source: Front Plant Sci. 2021 Sep 28;12:714763. doi: 10.3389/fpls.2021.714763 (PMC8505677; doi:10.3389/fpls.2021.714763)
Supplement: Supplementary file 1 [file Data_Sheet_1.docx]

Supplementary Material

# Supplementary Data 1. Taxonomic description of the *P. guajava* complex

*Psidium guajava* L.

Shrub or tree up to ca. 12 m high, subglabrous to densely appressed pubescent on young growth and lower leaf surfaces, the trunk smooth, light brown to light gray- green, with large flaky scales; hairs whitish, yellowish, or silvery, up to ca. 0.7 mm long, erect or appressed; young twigs quadrangular, slightly to strongly winged, often sulcate (at least when dry), densely to moderately appressed-pubescent, the older twigs at first scaly with longitudinal striations or fibers, eventually smooth with irregular scales falling as patches. LEAVES elliptic, oblong, elliptic-oblanceolate, elliptic- obovate, or lanceolate, 4.5–14 cm long, 2.4–7.5 cm wide, 1.6–4 times as long as wide, densely to sparsely appressed pubescent below, subglabrous except for puberulent midvein above; apex acute, acuminate, to rounded; base rounded to slightly cordate; petiole 2–5 mm long, 1–2 mm thick, channeled, densely pubescent to subglabrous; venation brochidodromous distally to eucamptodromous proximally, the midvein impressed above, prominent below, the lateral veins 9–22 prominent pairs, ascending at angle of ca. 45°, nearly straight, curving toward apex near the margin and connecting with the next lateral, the marginal vein not clearly present or arching between the laterals, the tertiary veins connecting the laterals in a ladder-like to reticulate pattern; blades coriaceous to submembranous, drying yellow-green, gray-green, to dark reddish brown. FLOWER BUDS subfusiform to pyriform, 9–14 mm long, sometimes strongly constricted near the midpoint, the hypanthium narrowly campanulate, barrel shaped or fusiform 4–6 mm long, the distal portion of bud more or less ovoid, sometimes strongly so with a conical apex, 4.5–9.5 mm long; indumentum pattern of buds with peduncles, hypanthium, and bracteoles sparsely to moderately appressed pubescent, the calyx without glabrous to sparsely pubescent (usually less densely covered than that hypanthium), the calyx within glabrous or densely pubescent, the petals, disk, and style glabrous; peduncles 1–3-flowered, 1–3.5 cm long, 1–1.5 mm thick, terete; bracteoles linear to narrowly triangular, 2–5 mm long. CALYX closed, tearing irregularly as the bud opens, persisting or falling in ca. 3 parts; petals obovate to elliptic, 13–22 mm long; disk 4–6 mm across; stamens 280–720, 7–15 mm long; anthers 0.7–1 mm long, with 1–7(–10) glands; style 10–15 mm long; ovary 3–6-locular; ovules 90–180 per locule, multiseriate. FRUIT globose to pyriform, 2–6(–8) cm long, green to yellow without, with pink, yellow, or white flesh, aromatic; seeds numerous, subreniform, 3–4 mm long, more or less smooth, the seed coat ca. 0.25 mm thick. 2n = 22.

*P. guineense* Sw.

Shrub or small tree up to about 6 m high, typically densely covered with reddish brown to yellowish gray velvety indumentum on the inflorescence and young growth but sometimes glabrous or nearly so, the trunk smooth to scaly; hairs simple, usually spreading, grayish to reddish brown, ca. 0.3–0.5 mm long; young twigs densely to moderately velutinous, or less often glabrous, compressed to terete in section, losing indumentum in about 1 year, usually not angled but sometimes grooved when young, vigorous shoots sometimes weakly angled, the older bark usually remaining more or less smooth, less often somewhat flaky or stringy. LEAVES elliptic, elliptic-oblong, obovate, 4–11.5 cm long, 2–8 cm wide, 1.3–2.4 times as long as wide, usually densely to moderately velutinous below, glabrous to covered with hairs along the midvein above, the margin entire; apex obtuse, rounded, or acute; base rounded to acute; petiole 4–12 mm long, 1.5–2 mm thick, channeled, densely to sparsely pubescent, rarely glabrous; venation brochidodromous to eucamptodromous distally, the midvein impressed or nearly flat above, prominent below, the lateral veins 5–10 pairs, ascending at an angle of ca. 45°, diminishing and looping near the margin to connect with the next lateral, a clear marginal vein not formed, the tertiary veins, connecting the laterals in a ladder-like to reticulate pattern; blades coriaceous, drying yellowish brown to reddish brown, concolorous to somewhat darker above, when dry often mottled and/or lustrous above. FLOWER BUDS pyriform, 8–15(–17) mm long, the hypanthium ellipsoid to obconic, 3.5–7 mm long, the distal portion of bud ellipsoid, subglobose, or ovoid, 4.5– 10 mm long; indumentum pattern of buds with all external surfaces moderately to densely pubescent (rarely subglabrous), the calyx pubescent without, but less densely so than hypanthium, distally pubescent within, the petals pubescent without, the disk sparsely pubescent (less often glabrous), the style glabrous; peduncles terete to compressed, 9–25(–30) mm long, 1–2 mm wide, uniflorous or triflorous, the branches of the dichasium when present 2–12 mm long; bracteoles narrowly triangular, ca. 2–3 mm long, caducous at about anthesis. CALYX closed completely, or with a terminal, pore-like opening at the apex, tearing longitudinally to the staminal ring, usually in 5 parts, these sometimes persisting until the fruit matures, the margin of calyx pore if present sinuate or with 5 small lobes; petals elliptic to obovate, 7–11 mm long; disk 4– 5 mm across; stamens 180–300, 7–10 mm long; anthers 1–3 mm long, more or less introrsely dehiscent, the glands in the connective 1 to over 50; style 8–10 mm long; ovary 3–5-locular; ovules 50–100 per locule, ca. 8-seriate. FRUIT subglobose to ellipsoidal, 1–3 cm long; seeds (22–)27–250 per fruit, 3–4 mm long. 2n = 44.

*P. guyanense* Pers.

Tree or shrub to 1-5 m high, minutely hirtellous on young growth; *hairs* mainly less than 0.1 mm long, erect, whitish to yellowish brown; *young twigs* minutely and sparsely hirtellous, sometimes appearing glabrous, drying dark reddish brown to light brown, the bark of older twigs smooth or flaky, often gland-dotted, the hairs persisting on first bark until it falls. LEAVES ovate, lanceolate, or elliptic, 6-13.5 cm long, 3-6.5 cm wide, (1.3-)1.8-2.6(-3) times as long as wide; *apex* acute to acuminate; *base* rounded, acute or cuneate; *petiole* 3-9 mm long, 1.5-2 mm thick, slightly to deeply channeled, minutely hirtellous; *venation* brochidodromous distally, eucamptodromous proximally, the midvein impressed or flat above, prominent below, the lateral veins 4-7, the marginal arching broadly distally, 0.5-3(-5) mm from margin, the tertiary venation dendritic; *blades* sometimes submembranous at anthesis, coriaceous at maturity, drying dark reddish brown. FLOWER BUDS 8-12 mm long, pyriform, the hypanthium obconic to campanulate, ca. 3.5-5 mm long, the distal portion of bud subglobose, 4-7 mm long, often wider than long, sparsely hirsutilose; *indumentum pattern of buds* with external surfaces sparsely to densely hirsutilose, the calyx often less densely so than adjacent hypanthium, the calyx within densely hirsutilose, the disk hirsutilose to pubescent, the style glabrous or with scattered hairs; *peduncles* 1-3-flowered, 4-20 mm long, 1-1.5 mm wide, compressed; *bracteoles* caducous before anthesis, perhaps small, narrowly triangular, those seen narrowly elliptic, 5-7 mm long. CALYX nearly closed (with a small terminal pore), or bowl-like with a large terminal pore, with a sinuate margin, tearing into 5-lobes at anthesis, these ca. 5 mm long, 3-5 mm wide; *petals* obovate to suborbicular, 6-9 mm long, glandular; *disk* including staminal ring 5-6 mm wide after anthesis; *stamens* 200-400, ca. 10 mm long, sparsely pubescent to glabrous; *anthers* oblong, 1-1.5 mm long, with 3-5 glands in the connective; *style* ca. 10 mm long, the stigma peltate, ca. ca. 1 mm wide; *ovary* 3-5 locular; *ovules* 50-75 per locule, the placenta protruding, sometime reflexed with ovules pointing towards center of flower. FRUIT oblong to suborbicular, to 5 cm long, the fruit wall 4-6 mm thick; seeds (2.5)3-5 mm long, 44-188, with rounded and flat sides.

*P. nutans* O. Berg.

Tree or shrub 1–5 m high, glabrous or sparsely appressed antrorsely pubescent on distal inner surface of calyx (rarely puberulent on young growth); hairs if present colorless, to ca. 0.5 mm long; young twigs reddish brown to blackish brown when dry, smooth with numerous darker glands, the bark of older twigs lighter brown or tan, falling as flakes. LEAVES elliptic to obovate, 5–17 cm long, 3–12 cm wide, 1.4–2.6 times as long as wide; apex acute, acuminate, obtuse to rounded, often with a cuspidate tip; base cuneate, obtuse, rounded, oblique, or subcordate; petiole shallowly channeled, 3–10 mm long, 1–2.5 mm thick; venation eucamptodromous proximally, brochidodromous distally, the midvein impressed proximally to nearly flat above, prominent below, the lateral veins 4–8(–10) pairs, leaving the midvein at an angle of 30–60°, nearly straight near midvein, arching distally towards apex, the marginal vein usually evident distally, arching mostly between 1–3 mm from the margin, the tertiary veins dendritic to ladder-like in pattern; blades coriaceous, often lustrous above and below, drying reddish to blackish brown, often mottled above with lighter spots when dry, the margin sometime crenulate in part. FLOWER BUDS pyriform, moderately to strongly constricted at ovary summit, 7–12(–14) mm long, the hypanthium ellipsoid to campanulate, 3–6 mm long, the distal portion of bud ovoid to subglobose, 4–6.5(–9.5) mm long; indumentum pattern of buds with all surfaces glabrous or essentially so; peduncles 1–3-flowered, 0.4–2 cm long, flattened, 1–2 mm wide, the branches of the dichasia 0.4–1 cm long; bracteoles narrowly triangular, ca. 2 mm long, caduceus at or before anthesis. CALYX closed, or with a small apical pore-like opening, with 5 minute lobes on the margin of the opening, tearing irregularly at anthesis, usually in 4–5 persistent pieces 4–8 mm long, the tears not cutting the staminal ring, glabrous to sparsely pubescent distally within; petals elliptic to obovate, 0.8–1.4 cm long; disk ca. 3.5 mm across; stamens 110–240, 6–10 mm long; anthers 1.2–2(–3) mm long, with a few to several glands in the connective; style 9–13 mm long, the stigma peltate, 0.5–0.8 mm across; ovary 3–5- locular; ovules 50–105 per locule, the placenta protruding, sometimes somewhat peltate. FRUIT globose, to subpyriform, 0.7–2 cm long; seeds numerous (50 in one fruit), subtriangular with rounded edges, 3–4 mm long.

*P. rostratum* McVaugh

Tree or shrub 1.8-15 m high, densely to moderately appressed pubescent to strigose on young growth and some floral structures, glabrescent with age, the trunk smooth, tannish or reddish; *hairs* whitish to tawny yellow, ca. 0.5 mm long; *young twigs* subterete, compressed or obscurely quadrangular, without clear wings, densely appressed pubescent to glabrous, drying dark reddish black to tan, dotted with small glands, the bark of older twigs scaly to smooth, generally light reddish brown. LEAVES elliptic, ovate, or oblong, 2.6-13.5 cm long, 2-4.8 cm wide, 1.3-2.6 times as long as wide, moderately strigose-pubescent to glabrous below, sparsely pubescent to glabrous above; *apex* rounded, acute, or acuminate, the tip sometimes apiculate; *base* rounded, acute, or acuminate; *petiole* channeled or not, 2-5 mm long, 1-1.5 mm thick, sparsely strigose to glabrous; *venation* brochidodromous (sometimes eucamptodromous proximally), the midvein impressed or sulcate proximally above, prominent below, the lateral veins 7-13 pairs, usually leaving the midvein at an angle greater than 45 degrees, arcing slightly upwards, up to ca. 0.25 mm wide, the marginal vein arching between the laterals, 1-10 mm from the margin, the tertiary veins forming a dendritic to ladder-like pattern between the laterals; *blades* submembranous to subcoriaceous, drying gray-green to blackish green or reddish brown, often covered with numerous amber colored protruding glands, these largest along the midvein. FLOWER BUDS often with a rostrate tip (rostrum), discounting the rostrum, broadly pyriform, 10-12 mm long, the rostrate tip 1-5 mm long when present, the hypanthium obconic, 3-4 mm long, the distal portion of bud globose, 7-8 mm long; *indumentum pattern of buds* with external surfaces sparsely pubescent to glabrous, or calyx and hypanthium densely puberulent without, the calyx sparsely to densely pubescent within, the petals glabrous or ciliate, sometimes densely glandular, the disc entirely glabrous or puberulent on staminal ring, the style glabrous; *peduncles* uniflorous, 0.2-4.5 cm long, ca. 1 mm wide, subterete; *bracteoles* caducous before anthesis, unknown. CALYX closed in bud, often prolonged as a rostrum 1-5 mm long, or open only as a small pore at the apex, tearing irregularly at anthesis, the tears sometimes cutting the staminal ring; *petals* obovate to suborbicular, ca. 1 cm long; *disk* within staminal ring 2-10 mm across; *stamens* ca. 600 or perhaps more, ca. 1 cm long; *anthers* ca. 1 mm long, with 0 or 1 gland in the connective, the gland reddish; *style* ca. 1 cm long; *ovary* 3-4-locular in flower seen; *ovules* 15-26, radiating from an elongate, slightly peltate placenta. FRUIT subpyriform to subglobose, 1.5-4 cm long, the wall 2-6 mm thick; seeds 4-12 (or perhaps more), 8-12 mm long, blackish or brown, smooth, with rounded with flat sides.

*P. rutidocarpum* G. Don.

Tree 5-11 m high, pubescent on young growth and lower leaf surfaces, the trunk "mottled reddish brown, peeling" (ex Smith); *hairs* reddish brown to whitish, usually curled, ca. 0.5 (-1) mm long; *young twigs* quadrangular, winged, moderately to densely pubescent, the bark falling in about 1 year, the older twigs more or less smooth or scaly, round, the scales gray, the inner bark dark reddish brown. LEAVES lanceolate, often narrowly so, 4-11 cm long, 1.4-3.4 cm wide, (2-)2.9-4.4 times as long as wide, the margin slightly revolute; *apex* attentuate-acuminate; *base* rounded to obtuse; *petiole* channeled, pubescent, 3-5 mm long, ca. 1.2 mm thick; *venation* mainly eucamptodromous, sometimes brochidodromous distally, the midvein impressed above, prominent below, the lateral veins 11-25 prominent pairs, usually impressed above, ascending at an angle of ca. 45 degrees, nearly straight but curving upwards near margin, the tertiary veins numerous (more than 10 per cm of lateral), connecting the laterals in more or less ladder-like pattern; *blades* coriaceous, drying dark reddish brown to dark gray-green. FLOWER BUDS unknown but probably similar to *P. guajava* or *P. guineense*; *indumentum pattern of buds* probably with external surfaces glabrous to moderately pubescent, the inner suface of the calyx densely covered with reddish brown hairs, the disc subglabrous; *peduncles* 5-23 mm long, 1-1.2 mm thick; *bracteoles* unknown. CALYX closed in bud, tearing irregularly, usually in 3 to 5 parts, tearing to staminal ring, but usually not cutting into it; *petals* unknown; *hypanthium* with ca. 10 poorly defined longitudinal ridges (in young fruits at least); *disk* ca. 5 mm across; *stamens* ca. 300; *anthers* unknown; *style* unknown; ovary 3-locular; *ovules* 25–40 ovules per locule. FRUIT subglobose, 1.5-2.5 cm long; seeds 65-90, ca. 2.5 mm long.
